# Case report: Glucose 6-phosphate-isomerase deficiency combine with avascular necrosis of bilateral femoral head

**DOI:** 10.3389/fped.2022.909752

**Published:** 2022-08-30

**Authors:** Zhenqi Song, Kongjian Wang, Djandan Tadum Arthur, Zhongwen Tang, Feng Xiang, Jie Wen, Sheng Xiao

**Affiliations:** Department of Pediatric Orthopedics, Hunan Provincial People's Hospital, The First Affiliated Hospital of Hunan Normal University, Changsha, China

**Keywords:** glucose phosphate isomerase deficiency, hemolytic disease, abnormal gait, femoral head, avascular necrosis

## Abstract

**Background:**

Glucose 6-phosphate-isomerase deficiency (GPI) is an uncommon autosomal recessive genetic disorder characterized by chronic asphoric hemolytic anemia, jaundice, and hepatospleenomegaly of varying degrees. Avascular necrosis of the femoral head in children may be caused by Legg-calve-perthes disease or hematological diseases. However, to date, there is no literature reporting on femoral head avascular necrosis as a complication of GPI.

**Case presentation:**

Herein we report a 6-year-old child admitted with no pain and abnormal gait in both lower extremities for 3 years, the patient received a genetic inspection and radiology test. Full-exon detection and Sanger sequencing verification were performed on the children and their parents C. 553T>A homozygous missense mutation (NM_ 001289790, F 185 I) was found in exon 6 of the GPI gene, which was inherited from parents. The radiology test showed avascular necrosis of the bilateral femoral head. The patient received traction and wore a spica splint every night and non-weight bearing hip joint rehabilitation every day for 12 months, after which, the gait of the femoral head of this patient improved significantly, and follow up radiation results showed the area of avascular necrosis of the femoral head had decreased.

**Conclusion:**

Careful investigation of GPI children with abnormal gait is recommended to avoid misdiagnosis, GPI combined with avascular necrosis of the femoral head should be considered as a differential diagnosis in GPI children with abnormal gait.

## Introduction

Glucose-6-phosphate-isomerase (GPI) deficiency is a rare autosomal recessive genetic disorder. The first case of GPI deficiency was described in 1968 at the College of Medicine by Baughan (1). The known clinical features of this GPI deficiency include a pale complexion, rapid heart rate, chronic asphalemic hemolytic anemia of varying degrees, jaundice, hepatosplenomegaly, and gallstone (2), which may be associated with giant cell proliferation, reticulocytosis, hyperferritinemia. A few cases have been reported with neuromuscular dysfunction, intellectual disability (3), male children with abnormal penile erection, and other clinical manifestations (4).

GPI deficiency is rarely reported, and the orthopedic complications of this disease are even rarer. Herein, we report a case of GPI deficiency combined with bilateral avascular necrosis of the femoral head.

## Case description

A 6-year-old boy patient was admitted to the hospital with the chief complaint of “Abnormal gait in both lower extremities for 3 years.” The child's family complained that his lower limbs had abnormal gait for 3 years without pain when he began to walk independently in 2018, he went to the local rehabilitation hospital for rehabilitation training, but the gait did not improve. The gait abnormality of both lower limbs has worsened recently. Jaundice and severe anemia appeared 6 h after birth, which improved after treatment. After that, anemia and yellowing of the skin occurred repeatedly, accompanied by hepatosplenomegaly. He underwent a splenectomy at the age of 4 due to splenomegaly. The child could sit alone at 1 year old, stand alone at 2 years old, and walk alone at 3 years old but fall easily. He can communicate with others normally, with no limitation in upper limbs. His father was healthy, his mother suffered from thalassemia.

Physical examination: T: 36.1°C, P: 104 times/min, R: 20 times/min, body weight 16.7 kg, clear consciousness, yellowish sclera, pale eyelids, lip mucosa, pale nail beds. No hyperemia of the pharynx, no enlargement of bilateral tonsils. Abdominal distension, 8 cm below the right costal margin of the liver, 7 cm below the xiphoid process, tough texture, without tenderness. No spinal deformity, knee reflex, and Achilles tendon reflex can be induced, pathological signs, and meningeal irritation signs were negative. The gait of the lower limbs was obviously abnormal, with claudication of the staggering gait, no obvious swelling, tenderness in both lower limbs, restricted movement of the hip joints, mainly restricted hip abduction and external rotation. The lower limbs were basically of equal length, and good blood supply to the extremities, good sensation and movement could be achieved.

**Figure 1:**
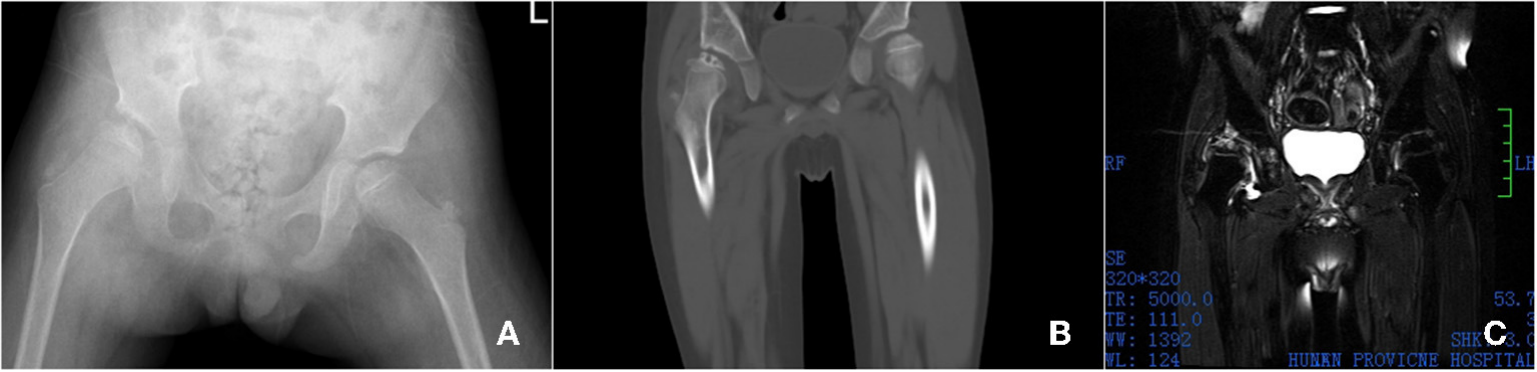
Imaging examination, X-ray shows the right femoral skull epiphysis becomes flat, smaller, irregular in shape, multiple cystic low-density lesions are seen inside, the right acetabulum is flat, and the right hip joint space is widened. The right femoral head shifted outwards and upwards **(A)**. CT shows the right femoral head epiphysis was flattened, several microcystic changes were observed on the upper edge, the right femoral head slightly shifted outwards and upward, and the right acetabulum was slightly flattened **(B)**. MRI showed that the right side Femoral skull epiphysis became flat and local bone collapsed. There were multiple small patchy T2WI hyperintensity shadows under the right femoral metaphysis and right acetabular articular surface **(C)**.

Laboratory examination: routine blood white blood cell count (WBC) 19.88 × 10 9/L, 2.62 × 1,012/L, hemoglobin (Hb) 85 g/L, serum ferritin (FRT) 6786.4 ng/ml, hematocrit 27.6%, platelet count 894 × 10 9/L coagulation function test: fibrin degradation product 31.30 μg/ml liver and kidney function test: total bilirubin 51.7 umol/L, direct bilirubin 17.1 umol/L, indirect bilirubin 34.6 umol/L, glutaminase 179.0 U/L, aspartate aminotransferase 89.72 u/L, alkaline phosphatase 188 U/L, r-glutamyl transpeptidase 81.5 U/L. Imaging examination: Pelvic ap film: the right femoral skull epiphysis becomes flat, smaller, irregular in shape, multiple cystic low-density lesions are seen inside, the right acetabulum is flat, and the right hip joint space is widened. The right femoral head shifted outwards and upwards (Figure 1A). CT results: the right femoral head epiphysis was flattened, several microcystic changes were observed on the upper edge, the right femoral head slightly shifted outwards and upward, and the right acetabulum was slightly flattened (Figure 1B). MRI results show right side femoral epiphysis became flat and local bone collapsed: there were multiple small patchy T2WI hyperintensity shadows under the right femoral metaphysis and right acetabular articular surface (Figure 1C).

## Diagnostic assessment

**Figure 2:**
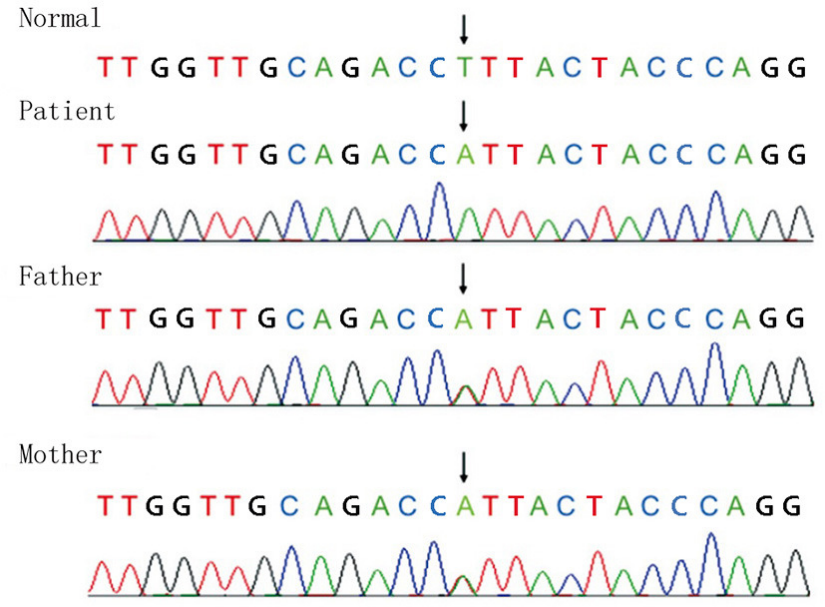
Full-exon detection and Sanger sequencing verification were performed on the children and their parents C. 553T>A homozygous missense mutation (NM_ 001289790, F 185 I) was found in exon 6 of the GPI gene, which was inherited from the parents.

Full-exon detection and Sanger sequencing verification were performed on the children and their parents C. 553T>A homozygous missense mutation (NM_ 001289790, F 185 I) was found in exon 6 of the GPI gene, which was inherited from parents (Figure 2). The mutation was highly likely to cause disease (correlation score of pathogenicity was 0.983) by POLY Phen 2 software. Provean software predicted that the mutation was a deleterious mutation affecting protein function (influence score was −4.995).

## Treatment and follow up

**Figure 3:**
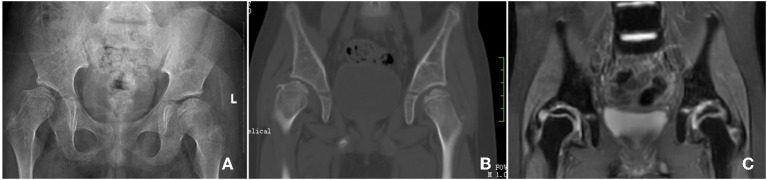
Follow up Imaging examination, X-ray shows the shape of the femoral head was remodeling **(A)**; the follow up CT result shows the microcystic area was replaced by normal bone tissue **(B)**; the follow up MRI result shows the multiple small patchy T2WI hyperintensity shadows under the right femoral metaphysis was disappear **(C)**.

The patient received traction and wore a spica splint every night and non-weight bearing hip joint rehabilitation every day for 12 months. After this period, the gait of the femoral head of this patient improved significantly and the follow up X-ray showed the shape of the femoral head was remodeling (Figure 3A). The follow up CT result showed the microcystic area was replaced by normal bone tissue (Figure 3B). The follow up MRI result showed that the multiple small patchy T2WI hyperintensity shadows under the right femoral metaphysis had disappeared (Figure 3C).

## Discussion

Despite recent research efforts, the etiology, clinical manifestations, and treatment of GPI deficiency are still poorly understood. The disease is essentially a hemolytic disease characterized by hereditary chronic non-spherical red blood cell hemolytic anemia.

Multiple blood transfusions can lead to progressive and pathological iron accumulation, resulting in transfusions of iron overload (5). The normal body iron storage is 3–4 g, and more than 20 g can cause damage to tissues and organs throughout the body. Excessive iron deposition can lead to a variety of terminal organ damage, including cardiomyopathy, liver dysfunction, anterior pituitary hormone deficiency, and diseases of the parathyroid, pancreas, and bone (6). Studies show iron overload leads to increased oxidative stress in a mouse model. After oxidative stress, RANKL-induced osteoclast formation was promoted, meanwhile, inflammatory changes were induced, bone remodeling, increased bone absorption was mediated, and bone cortical thinning caused bone loss (7). This may be an important reason for the necrosis of the femoral head on both sides in this case.

Legg-Calve-Perthes Disease (LCPD) is also a self-limited Disease in children. However, after the natural healing of the lesion, the necrotic femoral head is often left with a flat deformity, which is also called a flat hip. It is more common in unilateral femoral of children between 4 and 10 years old (8). The pathogenesis and pathological process are not clear yet. Childhood hematological avascular necrosis of the femoral head should be differentiated from LCPD. According to the X-ray features summarized by Iwegbu (9), hematological diseases with avascular necrosis of the femoral head have the following 5 manifestations: 1, subchondral sclerosis, the high-density area in the epiphyseal plate of the femoral head may be an early infarction; 2, perthes disease-like lesions, the sheet-like necrosis in the epiphyseal plate of the femoral head or the femoral head. When the epiphyseal plate of the femoral head is involved, a disease-like lesion similar to Perthes will appear, leading to 3, complete femoral head damage, which may appear as severe hip deformity or complete damage of the femoral head in the early stage, which can be partially reconstructed. 4, Central necrosis: the central part of the femoral head presents a cyst with varying sizes, originating from subchondral sclerosis or initial lesions. 5, Diffuse necrosis, involving necrosis of the entire femoral head, with multiple gouge-like changes, which could be a progressive manifestation of central necrosis.

Reviewing this case report, according to the patient's age, blood disease history, and imaging characteristics, it can be judged that this case of GPI combined with avascular necrosis of the femoral head is not caused by LCPD, but is caused by hematological disease-related avascular necrosis of the femoral head. It belongs to type 2 of the Iwegbu classification, which is similar to the lesions of Perthers' disease. We assume that the lack of glucose phosphate isomerase can make the red blood cells unable to be normally charged, resulting in the change of the shape of the red blood cell membrane, and in the case of relative acidosis, it will make the blood more viscous (4). Blood stasis, resulting in local tissue ischemia and hypoxia, causes increased anaerobic metabolism, tissue edema, and exudation increase resulting in a further increase in intraosseous pressure, continuous ischemia, hypoxia, and slow blood backflow, which can also induce blood hypercoagulation State (10). This may be one of the important reasons for avascular necrosis of the femoral head in this case.

Periodic blood transfusion and iron chelation therapy have significantly increased the life expectancy of patients with hemolytic disease, while the associated incidence of ischemic necrosis of the femoral head is increasing. Iron binds tightly to various proteins and enzymes in the body and can bind to hemoglobin to carry oxygen to tissues throughout the body. In hemolytic disease, a variety of reasons make red blood cells continue to destroy, dissolve, and release a large number of free iron, intestinal iron absorption capacity is limited, and the human body has no way to discharge iron, which can cause non-transfusions of iron overload. Multiple blood transfusions can lead to progressive and pathological iron accumulation, resulting in transfusions of iron overload (7). Normal body iron storage is 3–4 g, and more than 20 g can cause damage to tissues and organs throughout the body. Excessive iron deposition can lead to a variety of terminal organ damage, including cardiomyopathy, liver dysfunction, anterior pituitary hormone deficiency, and diseases of the parathyroid, pancreas, and bone (6). Some scholars have proposed a mouse model in which iron overload leads to increased oxidative stress. After oxidative stress, RANKL-induced osteoclast formation will be promoted, and meanwhile, inflammatory changes in the body can be induced. Through changes in bone remodeling, increased bone absorption will be mediated, and bone cortical thinning will result in bone loss (11). In a case report a child's serum ferritin (FRT) increased, the body of free iron significantly increased, meaning ferritin may be deposited in the femoral neck, resulting in the occlusion of bone microcirculation, increased intraosseous pressure, arteriolar compression, and bone loss, etc., leading to local blood supply insufficitude, hypoxia, and edema. This is one of the important reasons for the necrosis of the femoral head on both sides in this case.

There is no literature report treatment of GPI combined with bilateral femoral head necrosis, we can only refer to the treatment of Perthes disease for the treatment of femoral head necrosis in addition to the routine treatment of hemolytic disease. In non-surgical treatment, the patient is forbidden to bear weight, to reduce the load on the femoral head. They are then required to wear a spica splint during the night to increase the containment of acetabular. The brace must be worn for more than 2 years (12). Non-weight-bearing hip joint rehabilitation has also been reported to significantly improve joint, muscle function, and range of motion of the hip (12). In this case, we gave the patient a conservative treatment for 12 months, and the avascular necrosis of the femoral head of this patient was relieved and the function of hips remained at a good level.

There are several limitations in this study, first, we do not have direct evidence to prove avascular necrosis of the bilateral femoral head is caused by GPI, but we hope that we can accumulate some evidence for the research of GPI and femoral head necrosis in the future. Second, the follow up was not long enough, we will focus on this case and present the long term follow up results in the future.

## Conclusion

This is the first case report of a GPI deficiency patient with bilateral avascular necrosis of the femoral head among the literature on this subject. Careful investigation of GPI children with abnormal gait is recommended to avoid misdiagnosis, GPI combined with avascular necrosis of the femoral head should be considered a differential diagnosis in GPI children with abnormal gait.

## Data availability statement

The datasets for this article are not publicly available due to concerns regarding participant/patient anonymity. Requests to access the datasets should be directed to the corresponding author.

## Ethics statement

Written informed consent was obtained from the minor(s)' legal guardian/next of kin for the publication of any potentially identifiable images or data included in this article.

## Author contributions

ZS and KW drafted the initial manuscript, carried out the initial analyses, reviewed, and revised the manuscript. DA, ZT, and FX designed the data collection instruments, collected data, and critically reviewed the manuscript. JW and SX conceptualized and designed the study, coordinated and supervised data collection, reviewed, and revised the manuscript. All authors approved the final manuscript as submitted and agree to be accountable for all aspects of the work.

## Funding

This work was supported by Scientific Research Foundation of Hunan Provincial Education Department (21A0054).

## Conflict of interest

The authors declare that the research was conducted in the absence of any commercial or financial relationships that could be construed as a potential conflict of interest.

## Publisher's note

All claims expressed in this article are solely those of the authors and do not necessarily represent those of their affiliated organizations, or those of the publisher, the editors and the reviewers. Any product that may be evaluated in this article, or claim that may be made by its manufacturer, is not guaranteed or endorsed by the publisher.

## Abbreviations

GPI: Glucose 6-phosphate-isomerase
NLK: Neuroleukin
WBC: White blood cell count
Hb: Hemoglobin
FRT: Serum ferritin.

## References

[1] Baughan MA Valentine WN Paglia DE Ways PO Simons ER DeMarsh QB. Hereditary hemolytic anemia associated with glucosephosphate isomerase (GPI) deficiency–a new enzyme defect of human erythrocytes. Blood. (1968) 32:236–49. 10.1182/blood.V32.2.236.236 5672849

[2] Wong P Fuller PJ Gillespie MT Milat F. Bone disease in thalassemia: a molecular and clinical overview. Endocr Rev. (2016) 37:320–46. 10.1210/er.2015-1105 27309522

[3] Fermo E Vercellati C Marcello AP Zaninoni A Aytac S Cetin M. Clinical and molecular spectrum of glucose-6-phosphate isomerase deficiency. Report of 12 new cases. Front Physiol. (2019) 10:467. 10.3389/fphys.2019.00467 31133865 PMC6514191

[4] Goulding FJ. Priapism caused by glucose phosphate isomerase deficiency. J Urol. (1976) 116:819–20. 10.1016/S0022-5347(17)59030-8 1003664

[5] Grace RF Mark Layton D Barcellini W. How we manage patients with pyruvate kinase deficiency. Br J Haematol. (2019) 184:721–34. 10.1111/bjh.15758 30681718

[6] Kugler W Breme K Laspe P Muirhead H Davies C Winkler H. Molecular basis of neurological dysfunction coupled with haemolytic anaemia in human glucose-6-phosphate isomerase (GPI) deficiency. Hum Genet. (1998) 103:450–4. 10.1007/s004390050849 9856489

[7] Tsay J Yang Z Ross FP Cunningham-Rundles S Lin H Coleman R. Bone loss caused by iron overload in a murine model: importance of oxidative stress. Blood. (2010) 116:2582–9. 10.1182/blood-2009-12-260083 20554970 PMC2953890

[8] Set T Maras I Khan AS Ozdemir H. Management of Legg-Calve-Perthes disease with acupuncture: a case report. Acupunct Med. (2013) 31:105–7. 10.1136/acupmed-2012-010255 23234840

[9] Iwegbu CG Fleming AF. Avascular necrosis of the femoral head in sickle-cell disease. A series from the Guinea Savannah of Nigeria. J Bone Joint Surg Br. (1985) 67:29–32. 10.1302/0301-620X.67B1.3968138 3968138

[10] Beutler E Sigalove WH Muir WA Matsumoto F West C. Glucosephosphate-isomerase (GPI) deficiency: GPI elyria. Ann Intern Med. (1974) 80:730–2. 10.7326/0003-4819-80-6-730 4832160

[11] Rich MM Schoenecker PL. Management of Legg-Calvé-Perthes disease using an A-frame orthosis and hip range of motion: a 25-year experience. J Pediatr Orthop. (2013) 33:112–9. 10.1097/BPO.0b013e318281ab44 23389562

[12] Brech GC Guarnieiro R. Evaluation of physiotherapy in the treatment of Legg-Calvé-Perthes disease. Clinics (São Paulo). (2006) 61:521–8. 10.1590/S1807-59322006000600006 17187087

